# What Should We Do With People Who Cannot or Do Not Want to Be Protected From Neurotechnological Threats?

**DOI:** 10.3389/fnhum.2021.703092

**Published:** 2021-08-04

**Authors:** Silvia Inglese, Andrea Lavazza

**Affiliations:** ^1^Fondazione IRCCS Ca’ Granda, Ospedale Maggiore Policlinico, Milan, Italy; ^2^Department of Neuroethics, Centro Universitario Internazionale, Arezzo, Italy; ^3^University of Pavia, Pavia, Italy

**Keywords:** mental integrity, neurorights, extended mind, transhumanism, neurotechnologies, neurotechniques

## Abstract

Neurotechnologies can pose a threat to people’s privacy and mental integrity. Hence the proposal of establishing neurorights (Ienca and Andorno, 2017) and technical principles for the implementation of these rights (Lavazza, 2018). However, concepts such as “the extended mind” and what might be called “the post-human objection” can be said to challenge this protection paradigm. On the one hand, it may be difficult to outline the cognitive boundaries between humans and machines (with the consequent ethical and legal implications). On the other hand, those who wish to make strong use of neurotechnologies, or even hybridize with them, reject the idea that privacy and mental integrity should be protected. However, from the latter view, issues may arise relating to the protection of persons entering into relationships with posthumanist people. This article will discuss these scenarios as well as the ethical, legal, social, and political issues that could follow from them.

## Introduction: Protection From Neurotechnology Threats in Practice

In December 2020, the Chilean Senate approved a bill introducing a number of neurorights to be protected and a draft constitutional reform aimed at including the concept of protection from neurotechnology threats in the country’s new fundamental charter (Muñoz, 2019; Bosoer, 2021).

Specifically, the new law aims to: “Protect the physical and mental integrity of individuals, through the protection of the privacy of neuronal data, the right to autonomy or liberty of individual decision-making, and the right to fair access, without arbitrary discriminations, to those neurotechnologies that enhance mental capabilities.” It also establishes the need to “guarantee information to users of neurotechnologies regarding potential negative consequences and side effects, and the right to voluntarily control the functions of any devices connected to one’s brain.”

Indeed, neurorights have been recently proposed as new rights to be considered in addition to the already existing ones (Bublitz and Merkel, 2014; Ienca and Andorno, 2017; Yuste et al., 2017). These new rights might lead to an updated understanding of privacy, freedom of conscience, thought and speech, as well as the right to autonomy and self-determination. In fact, the advancement of neurotechnologies seems to open up new challenges to the protection of mental integrity, understood as the protection of people’s mental and cerebral functioning^1^. In this context we propose a specific definition of mental integrity, which may help clarify the discussion and avoid terminological misunderstandings.

*“Mental integrity” is the ability to formulate thoughts, judgments and intentions, make plans and implement them without direct external interference of any kind due to neurotechnology.*

Neurotechnologies are those techniques and instruments (for instance, brain scans, fMRI-based lie detection, brain computer interfaces, invasive and non-invasive brain stimulation, closed-loop brain implants) that allow direct or indirect access to a person’s cognitive system (also thanks to AI), understood as the neuronal correlates of one’s mental states (whether or not one’s mental states totally coincide with these brain correlates) (Bouthour et al., 2019; Kreitmair, 2019; Koenig-Robert and Pearson, 2020). This access is unprecedented since before the advent of current neurotechnologies only indirect inferences and manipulation could be made about an individual’s brain and mental states.

The need for neurorights seems to arise from the uniqueness of the potential threats that neurotechnologies pose to freedom of thought and mental integrity. Certainly, many sensitive data concerning the individual can be collected and disseminated, for example through social networks. However, the specificity of neurotechnologies is given by the unprecedented possibility of entering and violating the most intimate sphere of the person, the only one that could not otherwise be accessed (Just et al., 2017). The thought, the deepest convictions, the feelings, the beliefs that characterize all of us could soon be “read” or modified by the connection between brains and machines (think of the Neuralink project, cf., Fourneret, 2020).

This does not mean that resorting to neurotechnology itself poses threats to people. But some recent uses, documented mainly in China, seem to indicate how the state or private companies could abuse some neurotechnology for a new type of control of students or workers, requiring them to wear helmets that read their neuronal activations^2^. We do not yet have so powerful and precise neurotechnologies to make people puppets guided by external neural stimulation, however, the fast pace of research and application of new findings suggests anticipating the ethical reflection and consideration of legal aspects (Rickli and Ienca, 2021).

The enunciation of a right to mental integrity in the face of neurotechnologies is certainly the first step toward ensuring protection from potential risks, because it flags up the use of neurotechnology as potentially problematic in principle. But even those who acknowledge the potentially problematic aspect of neurotechnology disagree on how to provide effective protection of mental integrity. Some scholars have argued that it is not necessary to introduce new rights, but it is enough to apply international laws and treaties extensively. For example, it has been said that the European Convention on Human Rights, in particular through its article 8 (which protects the right to respect for one’s private life, family life, home, and correspondence), is perfectly adequate to deal with new forms of violation of privacy and even mental integrity (Ligthart, 2020).

In this respect, the dissent seems to be more theoretical or linked to elements of legal interpretation. Both supporters of new rights and advocates of revision or interpretation of existing ones share concerns about possible violations of fundamental rights and the need to protect them by punishing offenders. However, in our view, an underlying issue concerns the concrete protection to be adopted in relation to people’s mental integrity in the face of neurotechnologies specifically. Establishing rights may provide a basis for recognizing violations and imposing sanctions, but it does not in itself guarantee respect for mental integrity.

In this sense, one of us (AL) has formulated a technical principle:

*“The technical principle for the protection of mental integrity is a functional limitation that should be incorporated into any device capable of interfering with mental integrity”* (Lavazza, 2018).

It can be extended here in the following form: any neurotechnological device should: (a) incorporate systems that can find and signal any unauthorized detection and diffusion of brain data and any alteration of brain functioning; (b) be able to stop any unauthorized detection and diffusion of brain data and any alteration of brain functioning. This principle should not only concern specific devices, but act as a general (technical) operating principle shared by all interconnected systems that deal with decoding or intervening in brain activity.

A key point here is the idea of “unauthorized” detection, diffusion, and intervention. The individual should be previously and properly informed of any risks and instantly as possible have the device stopped when any threat to her mental integrity is being brought about.

Also, if we do not want to have a system that automatically blocks any interference, it can either only signal them, or it can be programmed as desired for one of the two functions (blocking and signaling). However, it might be difficult and expensive to insert the control functionality into each and every device. In this sense, a compromise should be found between cost and degree of protection, and this implies a complex interaction among regulators and manufacturers. This could also slow down the arrival of new devices on the market, limiting opportunities for consumers and freedom of enterprise for manufacturers. Of course, this example of ethics by design is only one of the possible ways of regulating the use of neurotechnologies and trying to provide protection from their potential risks (Kraft and Giordano, 2017; McCarthy-Jones, 2019; Rainey et al., 2020; Sommaggio and Mazzocca, 2020; Douglas and Forsberg, 2021).

## Protection From Neurotechnology Threats: Always, and for All?

Many medical uses of neurotechnologies necessarily affect the mental integrity of the subjects who use them to treat serious ailments like, for example, Parkinson’s disease or depression. One of the best known and most discussed cases is that of Deep Brain Stimulation (DBS), in consequence of which the patients’ identity or psychological continuity can be modified (Goering et al., 2017; Gilbert et al., 2018; Pugh et al., 2018). In such cases, the patient’s interest should come first, and it makes no sense to prohibit such interventions altogether. If the patient has signed an informed consent outlining all the possible side effects of the therapy and is able to stop the stimulation or require any necessary adjustments, then there is no need for the law to intervene in general. Such devices would not have to comply with the technical principle of protection of mental integrity.

However, we would like to deal with more controversial cases, which open up difficult scenarios that require reflection. The protection of mental integrity should in fact be as preventive as possible, as the values and rights at stake are truly of the utmost importance. We cannot allow people to be monitored or manipulated in the most essential features of their lives and personality. It is therefore reasonable to think about applying the technical principle of protection of mental integrity in a restrictive form, prohibiting any attempt at monitoring or manipulating people’s brain/mind activity, except for medical uses like those mentioned above^3^.

On the other hand, scenarios can be envisaged in which the use of neurotechnologies that may interfere with brain/mental functioning is not required for treatment but is desired by the subject concerned: this raises questions about the consent that should be required of the subject and the individual and social consequences of such use. We will now look in detail at three scenarios that exemplify situations of this kind and raise issues that do not yet seem to have adequate answers.

Obviously, the scenarios we describe do not cover all situations in which an individual may want to take the risk of a violation of their mental integrity. For example, recreational uses of neurotechnology could be viewed as more important than the risk to privacy. The attempt to obtain a cognitive enhancement that gives a competitive advantage could also be judged so relevant as to overcome all precautions aimed at protecting the freedom of thought. In these cases, devices that only signal the interference could be sold without restrictions, to avoid an excess of paternalism. However, from the perspective we defend here a certain amount of paternalism might be considered acceptable as we will explain the last section of the article.

### Extended Mind and Prevention of Alzheimer’s Disease

The founding example of the Extended mind thesis (EMT) is the case of Otto, an Alzheimer’s patient who writes down all the information that matters to him in a notebook, checking it whenever needed in order to find his way in the world (Clark and Chalmers, 1998). Otto’s situation is compared to that of Inga, a young woman who can rely on a perfectly efficient natural memory. EMT proponents argue that, from a cognitive point of view, there is no difference between the two, because a so-called principle of parity may apply.

If a part of the external world functions as a process that, had it taken place in our heads, we would not have hesitated to consider part of our cognitive process, then that part of the world is fully part of our cognitive process. This is an extension of the functionalist principle according to which mental states are identified by their causal-functional roles, without reference to their physical realizers. Nothing can exclude the possibility that they are located outside the skull and body; therefore, the mind can be extended into the world. It follows that Otto’s notebook is also part of Otto’s mind, which thus extends to the paper medium. The theory has no immediate ethical or pragmatic implications in the framework we are considering, but this would change if Otto’s notebook were replaced by some neuroprosthesis or a brain-machine interface (BCI).

For example, today one can obtain a very early diagnosis of the possible onset of Alzheimer’s disease (Ritchie et al., 2014, 2017). In these situations, an individual may wish to use, should the appropriate technology be available, a direct connection to a repertoire of digital memories or a real-time online support device to counteract their cognitive decline. However, if recourse to these tools were made before the symptoms of the illness developed, there could be possible alterations in the individual’s cerebral/mental integrity in the form of a change in their interaction with the world. Indeed, their psychological identity would probably be strongly influenced by these external aids – or “prompters” – in unprecedented ways.

The debate on the extended mind thesis is relevant here (Heersmink, 2017; Heinrichs, 2018). Scholars are in fact wondering what could and could not act as an external vehicle of the mind: they have introduced some constraints to avoid letting the theory become, like functionalism, too liberal in its ontology of the mind. The processes that can play the role of vehicles of cognition should always be available to the subject when needed, be easily accessible and be transparent, i.e., they should give an output that the mental system uses directly, without filters, as if it came from an internal part of it.

This has implications for the topic of protection. Should uses of neurotechnologies such as those mentioned above be subject to adjustment when they are not required as a therapy to restore a previous condition? To what extent are those neurotechnologies really “integrated” into the individual and to what extent do they constitute an alteration of their mental integrity, even if accepted by the user? It is possible that different neurotechnologies should be subject to different assessments and regulations. In the conclusion of this article, after having exposed the other scenarios, we will suggest some general guidelines.

### A Society of Enhanced Humans?

Advanced societies seem to require an increasing degree of cognitive capacities in order to manage complex processes on both local and global scales, as well as to deal with planetary emergencies such as climate change, the destruction of the natural environment and the accelerated consumption of natural resources (cf., Lavazza, 2019a; Lavazza and Reichlin, 2019). In the face of these challenges, it has been argued (Rindermann, 2018) that the availability of cognitive skills, especially in the Western world, may decrease due to ongoing demographic trends. In many countries, lower birth rates and longer life expectancy mean that there are increasing numbers of individuals with declining memory, processing speed, attention, creativity, and overall capacity for innovation. Another associated phenomenon is that more educated and cognitively efficient people tend to have fewer or no children.

In this sense, it is plausible to assume that progressive aging causes a decrease in the cognitive abilities of society as a whole. Although we are seeing an increase in the number of fit and active septuagenarians and octogenarians, there is evidence that in general “the normal aging process is associated with declines in certain cognitive abilities, such as processing speed and certain memory, language, visuospatial, and executive function abilities” (Harada et al., 2013).

In the face of this trend, one could hypothesize introducing widespread forms of neurocognitive enhancement through neurotechnology, such as the various forms of non-invasive brain stimulation available today (Woods et al., 2016; Lavazza, 2019b). This would be a way of enabling society as a whole to better cope with the growing challenges outlined above through new tools made available by research^4^. As is well known, some philosophers have argued that human beings are unfit to deal with at least two types of serious problems “generated by the existence of modern scientific technology: the threats of weapons of mass destruction, especially in the hands of terrorist groups, and of climate change and environmental degradation” (Persson and Savulescu, 2012, p. 1). Consequently, cognitive enhancement, which is considered a prerequisite for moral improvement, could be strongly recommended, encouraged, or even become compulsory.

How can these demands be reconciled with the idea that people are entitled to full respect for their mental integrity? A cognitive and moral enhancement of the kind described would certainly lead to an alteration of the identity and psychological continuity of individuals, even if it were directed toward personal or collective improvement, or the protection of society in the face of more or less imminent dangers. The risk of radical paternalism, which would allow some decision-makers to choose for many or all to what degree mental integrity could be sacrificed for a supposedly greater good, is evident. However, the very idea of neurorights as human rights seems to conflict with the view that, even under a democratic process, individual guarantees established as basic and inviolable could ever be waived.

### Radical Posthumanist Fusion

A more extreme case is that of posthumanists or transhumanists, who wish to hybridize or merge with digital devices and instruments to varying degrees (Nayar, 2018; Benedikter and Fathi, 2019; Lee, 2019). This can require freely sharing all of one’s brain data or allowing a machine to make decisions in relevant domains of one’s life. Such an existential vision wishes to go beyond the boundaries and limits of human beings with their naturally variable endowments. We cannot exclude that some individuals may not be at all interested in protecting their own mental integrity: for them, violations of privacy or alterations in the functioning of their mind/brain would be to their advantage, or at least not to their serious disadvantage.

In these cases, what regulations should be put in place? Should we allow selling devices without security controls for those who want to have their mind enhanced or modified? Or should the State introduce paternalistic rules in order to protect the citizens from unpredictable damages to their mental integrity? As we shall see in the Conclusion, there may be an argument to be made in this direction. There is, however, another important circumstance to consider. Indeed, transhumanists who have hybridized with some neurotechnology may raise issues that are relevant to *all* citizens and therefore cannot pass the harm principle test. According to this principle, which values individual autonomy, each individual should be free to choose the conduct they prefer as long as it does not harm others.

Two types of issues seem to arise in relation to those who choose a “deep interaction” with neurotechnologies and digital machines. The first type concerns ethical and legal consequences. On the one hand, those who are willing to sacrifice their mental integrity might gain an (unfair?) competitive advantage over all other individuals acting under the principle of protection, which thus would not fully benefit from the potential of neurotechnologies (e.g., those aimed at radical enhancement, which can also affect one’s identity and psychological continuity). On the other hand, when we consider cyborgs “merged” with self-driving cars or implanted closed-loop devices capable of provoking automatic behavioral responses to changing circumstances, who would be legally responsible for their conduct, should it harm others? The ontology of extended mind would be relevant here, so as to establish where the individual’s mind/brain “ends” and where the machine, which cannot be charged with a moral or legal violation, “begins” (Gallagher, 2018; Kiverstein et al., 2019).

The second type of issue is even more relevant to the topic of protection from neurotechnology threats itself. Those who do not place limits on the neurotechnologies they use may become a vehicle for violating the neurorights of others. This could happen, for instance, with tools for detecting the emotions or thoughts of others – tools used as enhancers of social interaction skills, which, however, violate mental/cerebral privacy. So how should one deal with posthumanists and transhumanists? Should an opt-out clause be introduced in generalized forms of protection? But that would leave us with the possible “side effects” produced by those who renounce their neurorights. These scenarios, in our opinion, deserve careful consideration in the field of protection from neurotechnology threats and its concrete declinations, including legislative forms.

## Conclusion: A Call for Caution

The cases we have described challenge the simple affirmation of new neurorights and their implementation through a technical principle of prevention. How should we deal with non-clinical or quasi-clinical cases?^5^ Should we just make sure people give their simple informed consent to all of this? Or should we introduce a stricter form of informed consent?

Here we put forward an argument for some kind of precautionary principle to consider neurorights, at least in a circumscribed version that excludes clinical uses, as inalienable and irrevocable. It is not a question of introducing paternalistic prescriptions in the belief that people do not know how to choose what is best for them. Rather, in the face of increasingly powerful and invasive neurotechnologies, we may find ourselves in situations so new and remarkable that no one could really be aware of their consequences for one’s identity and psychological continuity.

In this sense, the appropriateness of preventing individuals from completely disposing of their mental integrity as they wish is based on that, once implemented the neurotechnology, it might be impossible to “go back.” This could happen for purely technical reasons: some implants may not be able to be removed or switched off. Most of all, mental integrity is what characterizes us humans and gives us a dynamic anchorage to reality. If a radical and sudden change takes place in this domain, we may no longer have the ability to make a comparison with the previous situation, or we may no longer have the resources to return to the previous situation, because it was lost in the neurotechnological transition.

To some extent, the unavailability of brain data has also been introduced in the Chilean legislation, which equates brain data to organ donation and transplantation. In this sense, the new bill states that brain data are highly sensitive elements about one’s most intimate personal sphere, something about which one has a unique first-person perspective and so an authorship that should not be violated. Therefore, brain data cannot be given away for any reason, just as organs cannot be sold (at least in most legislations).

Based on what has been said so far, the following synthetic guidelines can be proposed. *Ad hoc* international conventions (implemented by specific national laws) should introduce neurorights related to the potential threats posed by the use of neurotechnologies. Resorting to international conventions is suggested because national legislation would be easily circumvented. These neurorights should not only be spelled out but should also include rules for practical implementation. Here we propose a technical protection principle devised by introducing specific techniques in all devices produced and offered for sale.

On the market-side, all manufacturers should adapt to these standards, producing and placing on sale only neurotools that incorporate, as far as technically possible, devices capable of signaling and blocking unauthorized interference on the user’s mental integrity.

On the consumer-side, individuals should only be able to access tools that ensure the absolute protection of coded neurorights, other than medical uses that should be certified and authorized with detailed procedures. The possibility remains open to allow, with special informed consent, the use of devices that signal but do not automatically stop any violations of the user’s privacy and mental integrity.

In this article we have looked at cases that can be taken to be still marginal or somewhat futuristic but are likely to become relevant quite soon. These issues certainly require further conceptual clarification and ethical evaluation. So, the task of scientific and neuroethical reflection is to begin to address them in greater depth.

## Data Availability Statement

The original contributions presented in the study are included in the article/supplementary material, further inquiries can be directed to the corresponding author.

## Author Contributions

Both authors listed have made a substantial, direct and intellectual contribution to the work, and approved it for publication.

## Conflict of Interest

The authors declare that the research was conducted in the absence of any commercial or financial relationships that could be construed as a potential conflict of interest.

## Publisher’s Note

All claims expressed in this article are solely those of the authors and do not necessarily represent those of their affiliated organizations, or those of the publisher, the editors and the reviewers. Any product that may be evaluated in this article, or claim that may be made by its manufacturer, is not guaranteed or endorsed by the publisher.

## Footnotes

1 For reasons of space, here we must refrain from addressing some relevant issues. One concerns the complex distinction and relation between brain and mind. When necessary, we will use the intuitive term mind/brain and the corresponding adjectives.

2 See for instance, S. Chen, “Forget Facebook leak”: China is mining data directly workers’ brains on an industrial scale, *South China Morning Post*, April 29, 2018, https://www.scmp.com/news/china/society/article/2143899/forget-facebook-leak-china-mining-data-directly-workers-brains; T. F. Chan, China is monitoring employees’ brain waves and emotions -and the technology boosted one company’s profits by $315 million, *Business Insider*, May 1, 2018, https://www.businessinsider.com/china-emotional-surveillance-technology-2018-4?IR=T.

3 The issue concerning the medical uses of neurotechnologies would deserve a more in-depth discussion that we cannot develop here.

4 We will not dwell here on the effectiveness of current forms of non-invasive brain stimulation or other techniques, but we are aware that there are currently no devices available that can provide radical enhancement.

5 But one should consider how complex it is to draw a line between care and enhancement, and how large the gray area between the two can be.

## References

[B1] Benedikter R. Fathi K. (2019). The future of the human mind: techno-anthropological hybridization? *Challenge* 62 77–95. 10.1080/05775132.2018.1560943

[B2] Bosoer L. (2021). *Opinion: Chile at the Forefront of Neurorights Protection.* Available online at: https://blogs.eui.eu/latin-american-working-group/opinion-chile-at-the-forefront-of-neurorights-protection (accessed June 30, 2021).

[B3] Bouthour W. Mégevand P. Donoghue J. Lüscher C. Birbaumer N. Krack P. (2019). Biomarkers for closed-loop deep brain stimulation in Parkinson disease and beyond. *Nat. Rev. Neurol.* 15 343–352. 10.1038/s41582-019-0166-4 30936569

[B4] Bublitz J. C. Merkel R. (2014). Crimes against minds: on mental manipulations, harms and a human right to mental self-determination. *Crim. Law Philos*. 8 51–77. 10.1007/s11572-012-9172-y

[B5] Clark A. Chalmers D. (1998). The extended mind. *Analysis* 58 7–19.

[B6] Douglas T. Forsberg L. (2021). “Three rationales for a legal right to mental integrity,” in *Neurolaw: Advances in Neuroscience, Justice & Security*, eds Ligthart S. van Toor D. Kooijmans T. Douglas T. Meynen G. (London: Palgrave Macmillan), 179–201. 10.1007/978-3-030-69277-3_8

[B7] Fourneret É (2020). The hybridization of the human with brain implants: the neuralink project. *Camb. Q. Healthc. Ethics* 29 668–672. 10.1017/s0963180120000419 32892780

[B8] Gallagher S. (2018). The extended mind: state of the question. *South. J. Philos.* 56 421–447. 10.1111/sjp.12308

[B9] Gilbert F. O’Brien T. Cook M. (2018). The effects of closed-loop brain implants on autonomy and deliberation: what are the risks of being kept in the loop? *Camb. Q. Healthc. Ethics* 27 316–325. 10.1017/s0963180117000640 29509128

[B10] Goering S. Klein E. Dougherty D. D. Widge A. S. (2017). Staying in the loop: relational agency and identity in next-generation DBS for psychiatry. *AJOB Neurosci.* 8 59–70. 10.1080/21507740.2017.1320320

[B11] Harada C. N. Love M. C. N. Triebel K. L. (2013). Normal cognitive aging. *Clin. Geriatr. Med.* 29 737–752. 24094294 10.1016/j.cger.2013.07.002 PMC4015335

[B12] Heersmink R. (2017). Distributed selves: personal identity and extended memory systems. *Synthese* 194 3135–3151. 10.1007/s11229-016-1102-4

[B13] Heinrichs J. H. (2018). Neuroethics, cognitive technologies and the extended mind perspective. *Neuroethics* 14 59–72. 10.1007/s12152-018-9365-8

[B14] Ienca M. Andorno R. (2017). Towards new human rights in the age of neuroscience and neurotechnology. *Life Sci. Soc. Policy* 13:5. 10.1186/s40504-017-0050-1 28444626 PMC5447561

[B15] Just M. A. Pan L. Cherkassky V. L. McMakin D. L. Cha C. Nock M. (2017). Machine learning of representations of suicide and emotion concepts identifies suicidal youth. *Nat. Hum. Behav.* 1 911–919. 10.1038/s41562-017-0234-y 29367952 PMC5777614

[B16] Kiverstein J. Farina M. Clark A. (2019). *The Extended Mind, Oxford Bibliographies Online.* New York: Oxford University Press.

[B17] Koenig-Robert R. Pearson J. (2020). Decoding nonconscious thought representations during successful thought suppression. *J. Cogn. Neurosci*. 32 2272–2284. 10.1162/jocn_a_01617 32762524

[B18] Kraft C. J. Giordano J. (2017). Integrating brain science and law: neuroscientific evidence and legal perspectives on protecting individual liberties. *Front. Neurosci*. 11:621. 10.3389/fnins.2017.00621 29167633 PMC5682320

[B19] Kreitmair K. V. (2019). Dimensions of ethical direct-to-consumer neurotechnologies. *AJOB Neurosci*. 10 152–166. 10.1080/21507740.2019.1665120 31642755

[B20] Lavazza A. (2018). Freedom of thought and mental integrity: the moral requirements for any neural prosthesis. *Front. Neurosci*. 12:82. 10.3389/fnins.2018.00082 29515355 PMC5825892

[B21] Lavazza A. (2019a). The two-fold ethical challenge in the use of neural electrical modulation. *Front. Neurosci.* 13:678. 10.3389/fnins.2019.00678 31316345 PMC6611379

[B22] Lavazza A. (2019b). Transcranial electrical stimulation for human enhancement and the risk of inequality: prohibition or compensation? *Bioethics* 33 122–131. 10.1111/bioe.12504 30157289

[B23] Lavazza A. Reichlin M. (2019). Introduction: moral enhancement. *Topoi* 38 1–5. 10.1093/acprof:oso/9780198707592.003.0001 33782627

[B24] Lee N.(ed.) (2019). *The Transhumanism Handbook.* Cham: Springer.

[B25] Ligthart S. (2020). Freedom of thought in Europe: do advances in ‘brain-reading’technology call for revision? *J. Law Biosci*. 7:lsaa048. 10.1093/jlb/lsaa048 34221423 PMC8249165

[B26] McCarthy-Jones S. (2019). The autonomous mind: the right to freedom of thought in the twenty-first century. *Front. Artif. Intell.* 2:19. 10.3389/frai.2019.00019 33733108 PMC7861318

[B27] Muñoz J. M. (2019). Chile–right to free will needs definition. *Nature* 574:634. 10.1038/d41586-019-03295-9 31664207

[B28] Nayar P. K. (2018). *Posthumanism.* New York, NY: John Wiley & Sons.

[B29] Persson I. Savulescu J. (2012). *Unfit for the Future: The Need for Moral Enhancement.* Oxford: Oxford University Press.

[B30] Pugh J. Pycroft L. Maslen H. Aziz T. Savulescu J. (2018). Evidence-based neuroethics, deep brain stimulation and personality-deflating, but not bursting, the bubble. *Neuroethics* 10.1007/s12152-018-9392-5 PMC8568854 34790274

[B31] Rainey S. McGillivray K. Akintoye S. Fothergill T. Bublitz C. Stahl B. (2020). Is the European data protection regulation sufficient to deal with emerging data concerns relating to neurotechnology? *J. Law Biosci.* 7:lsaa051. 10.1093/jlb/lsaa051 PMC8355473 34386243

[B32] Rickli J. M. Ienca M. (2021). “The security and military implications of neurotechnology and artificial intelligence,” in *Clinical Neurotechnology Meets Artificial Intelligence: Philosophical, Ethical, Legal and Social Implications*, eds Friedrich O. Wolkenstein A. Bublitz C. Jox R. J. Racine E. (Cham: Springer), 197–214. 10.1007/978-3-030-64590-8_15

[B33] Rindermann H. (2018). *Cognitive Capitalism: Human Capital and the Wellbeing of Nations.* Cambridge: Cambridge University Press.

[B34] Ritchie C. Smailagic N. Noel-Storr A. H. Takwoingi Y. Flicker L. Mason S. E. (2014). Plasma and cerebrospinal fluid amyloid beta for the diagnosis of Alzheimer’s disease dementia and other dementias in people with mild cognitive impairment (MCI). *Cochrane Database Syst. Rev.* 2014:CD008782. 10.1002/14651858.CD008782.pub4 24913723 PMC6465069

[B35] Ritchie C. Smailagic N. Noel-Storr A. H. Ukoumunne O. Ladds E. C. Martin S. (2017). CSF tau and the CSF tau/ABeta ratio for the diagnosis of Alzheimer’s disease dementia and other dementias in people with mild cognitive impairment (MCI). *Cochrane Database of Syst. Rev.* 3:CD010803. 10.1002/14651858.CD010803.pub2 28328043 PMC6464349

[B36] Sommaggio P. Mazzocca M. (2020). “Cognitive liberty and human rights,” in *Neuroscience and Law. Complicated Crossings and New Perspectives*, eds D’Aloia A. Errigo M. C. (Cham: Springer), 95–111.

[B37] Woods A. J. Antal A. Bikson M. Boggio P. S. Brunoni A. R. Celnik P. (2016). A technical guide to tDCS, and related non-invasive brain stimulation tools. *Clin. Neurophysiol.* 127 1031–1048. 10.1016/j.clinph.2015.11.012 26652115 PMC4747791

[B38] Yuste R. Goering S. Arcas B. A. Y. Bi G. Carmena J. M. Carter A. (2017). Four ethical priorities for neurotechnologies and AI. *Nature* 551 159–163. 10.1038/551159a 29120438 PMC8021272

